# The use of intraventricular vancomycin in subacute brain abscess in an adolescent male: A case report

**DOI:** 10.2478/jccm-2024-0046

**Published:** 2025-04-30

**Authors:** Tomas Leng, Ibrahim Serhat Karakus

**Affiliations:** Department of Pediatric and Adolescent Medicine, Mayo Clinic, Rochester, MN, USA; Department of Anesthesiology and Perioperative Medicine, Mayo Clinic, Rochester, MN, USA

**Keywords:** brain abscess, intraventricular vancomycin, pediatrics, neurocritical care, case report

## Abstract

**Introduction:**

Brain abscess is a serious condition in children, leading to rapid deterioration, and permanent neurological damage associated with significant morbidity and mortality. Current management protocols for brain abscesses focus on intravenous antibiotics and surgical excision and drainage.

**Case Presentation:**

A 12-year-old adolescent male who had headache and photophobia and was diagnosed with multiple brain abscesses and was refractory to conventional medical and neurosurgical intervention. A single dose of 10 mg vancomycin was administered through endo-ventricular drain, resulting in resolution of abscesses and alleviation of symptoms.

**Conclusion:**

We describe the first instance of intraventricular vancomycin use in the pediatric age group for the treatment of multiple brain abscesses. Given the variability in dosing reported in the literature, our case report warrants further studies to standardize dosage for this rare intervention.

## Introduction

Brain abscess (BA) is a rare condition in children, associated with significant morbidity and mortality. Symptomatology varies based on the location, extent, pathogen, and host immune status. They include fever, headache, seizures, focal deficits and altered level of consciousness. Complications include meningitis, cyst rupture, intraventricular rupture of brain abscess (IVROBA), herniation, and death. Chronic sequelae include epilepsy, motor and audiovisual deficits, and hydrocephalus [1, 2].

Investigations for BA include cell counts, inflammatory markers, imaging, cerebrospinal fluid (CSF) analysis, and cultures of blood and CSF. Intravenous (IV) antibiotics and surgical excision of abscess are the mainstay of management [1]. Intraventricular antibiotics, while indicated in children with ventriculoperitoneal shunt infections and meningitis, are not considered standard management in pediatric brain abscesses [3]. No prior existing guidelines or documented cases of intraventricular vancomycin used for BA in pediatric age group. We report the first use of intraventricular vancomycin to treat multiple brain abscesses in a pediatric patient.

## Case presentation

A previously healthy 12-year-old presented with a 10-day history of fever, headache and photophobia. Medical attention was sought prior, with symptomatic care advised. A lumbar puncture revealed cloudy CSF and elevated opening pressure of 30 cm H_2_O (reference range 7–18 cm H_2_O). Serum CRP was 27 mg/L (reference range < 5 mg/L) and leukocytes were 13000 /uL (reference range 3600 – 9100 /uL).

**Fig. 1.**
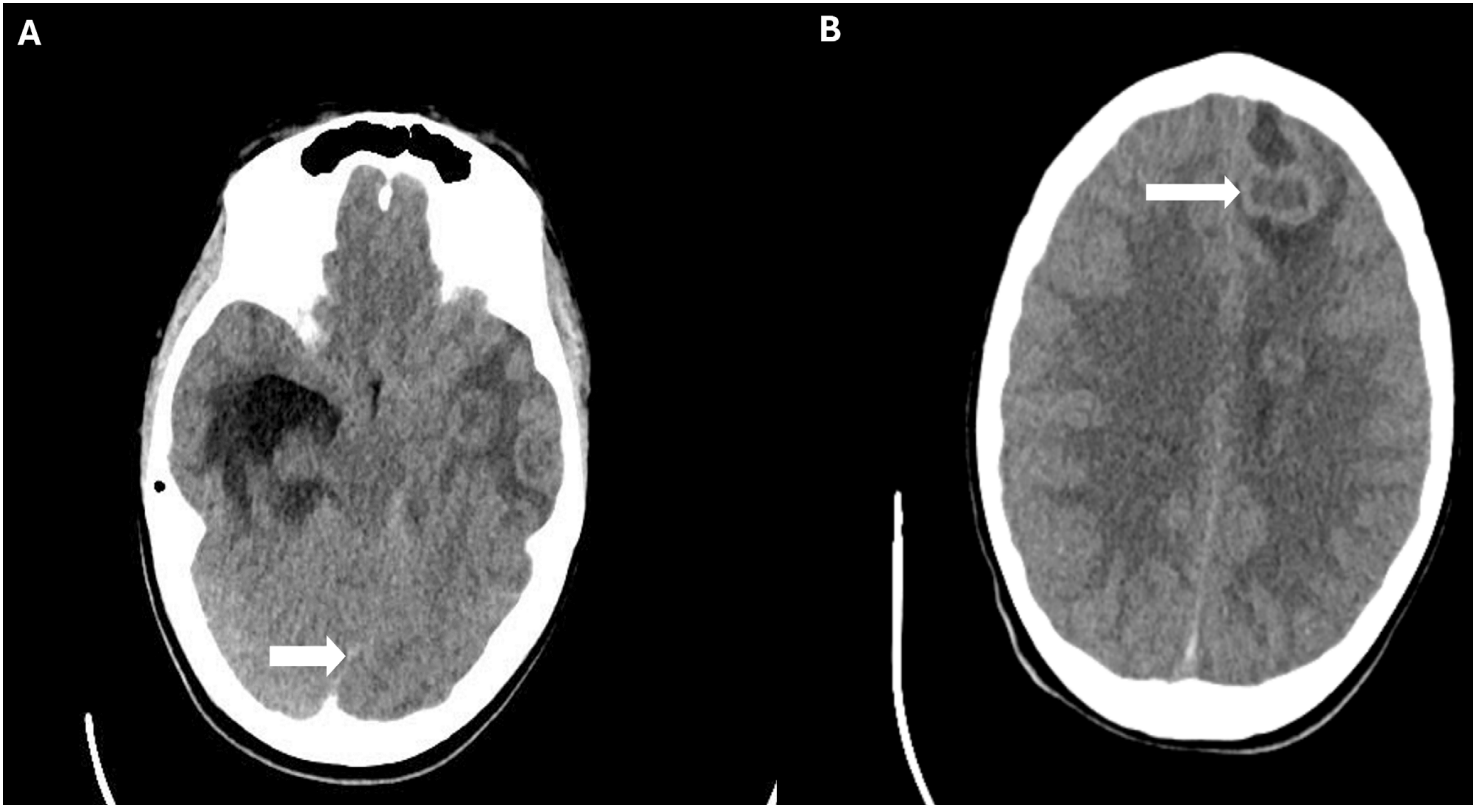
A: Axial head CT image showing the midline shift (white arrow); B: Axial head CT image showing multiple brain abscesses.

On transfer to our facility for presumptive meningitis, he had fever (Temperature-38.1 Celsius), bradycardia (heart rate between 50–60/minute), and tachypnea (respiratory rate of 28/min). Neurological examination was notable for neck stiffness and a positive Kernig’s sign. IV fluids, vancomycin, ceftriaxone, and acyclovir were started. His condition deteriorated, marked by worsening mental status, severe bradycardia and hypertension. He was admitted to the PICU, intubated, and received dexamethasone and mannitol for raised intracranial pressure (ICP), which did not elicit a response. A head CT revealed multiple supratentorial and infratentorial brain abscesses, midline shift, and hydrocephalus (Figure 1A and 1B).

Neurosurgery was consulted and performed a left-lateral endo-ventricular drain (EVD) insertion and a right temporal cystectomy. CSF cultures grew pansusceptible *Streptococcus intermedius*. Despite antibiotic therapy and periodic CSF drainage, persistently elevated ICP of up to 60 mmHg were noted. Given risk of imminent herniation, additional frontal and occipital EVDs were placed. A small volume of pus was observed upon the frontal drain insertion.

**Fig. 2.**
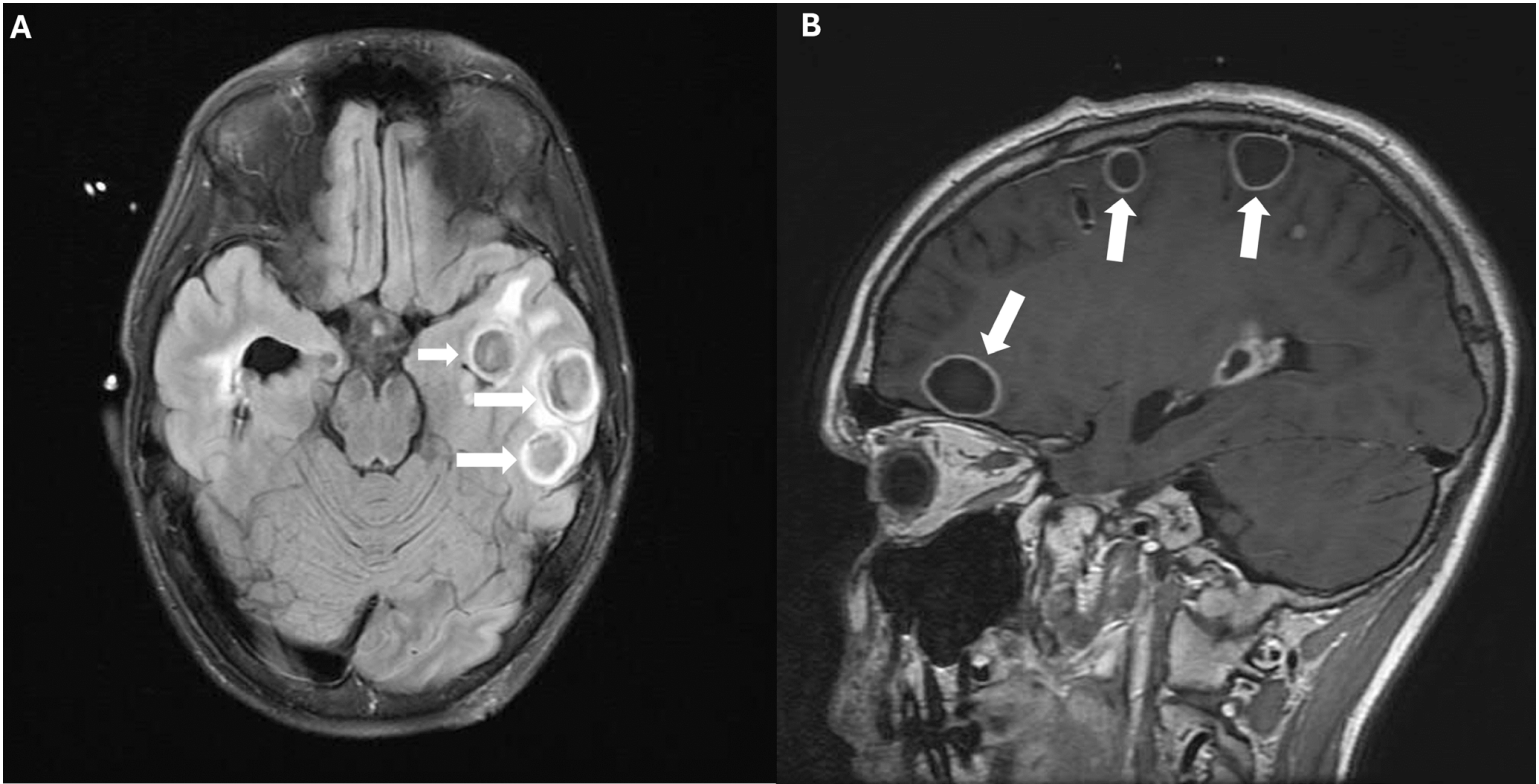
A and B Axial MRI images demonstrating resolving brain abscesses. White arrows highlight the reduced size of abscesses

Due to radiologic progression and the lack of clinical improvement, infectious disease specialists were consulted. They recommended administering intraventricular vancomycin due to subtherapeutic systemic vancomycin trough level. Neurosurgery administered a single dose of 10 mg vancomycin in 1 mL of normal saline via EVD under sedation. Following this, he remained afebrile. ICP settled in the 20’s. Subsequent CSF cultures were sterile. Repeat MRI showed resolving abscesses (Figure 2A and 2B). Sedation was weaned and he was extubated.

Blood cultures were negative. Echocardiography showed no structural heart disease. A full-body CT showed bilateral hilar and mediastinal lymphadenopathy; and calcified granulomas in the right lung, liver, and spleen; most likely due to prior histoplasmosis. Histoplasma serology with titers of 1:8 was suggestive of prior infection. An immune workup did not diagnose immunodeficiency. However, a lymphocyte proliferation panel displayed significantly decreased CD45+ and CD3 T-cell responses.

Following recovery, EVDs were given trial clamps, followed by serial CT which demonstrated lateral ventricular dilation and leftward shift. Given continued fluctuations in ICPs and ventriculomegaly, he underwent ventricular fenestration surgery. ICPs returned to normal and EVDs were removed. He was discharged after 34 days on IV ceftriaxone and oral metronidazole for 6 weeks. On follow-up four months later, he reported no symptoms.

## Discussion

In summary, this patient presented with multiple BA and raised ICP, refractory to standard management. EVD insertion was significant for purulent drainage, suggesting extensive ventriculitis versus intraventricular rupture of brain abscess (IVROBA), a dreaded complication of BA. The proximity of some of the abscesses to the ventricle, possible ependymal enhancement seen in the ventricular wall adjoining the abscesses, and the presence of debris in the lateral ventricles may indicate a component of IVROBA. Given the continued decline and risk of imminent uncal herniation, a single dose of 0.167 mg/kg intraventricular vancomycin was administered, following which ICP stabilized and subsequent CSF cultures were sterile.

CSF cultures grew *Streptococcus intermediu*s and investigations were consistent with prior Histoplasmosis without active signs of infection. Two prior reports describe multiple brain abscesses with *Streptococcus intermedius* concurrent with evidence of prior histoplasmosis, either by serology or through a chest CT [4, 5]. Neither proved histoplasma as an implicated pathogen, or associated lymphadenopathy and calcifications as a possible nidus of infection causing hematogenous spread to the brain. Similarly, we can only speculate if this was the case with our patient.

A lymphocyte proliferation panel revealed decreased CD45+ and CD3 T-cell responses suggesting possible decreased defense mechanisms, and increased susceptibility to opportunistic infections. Our patient received a single dose of 9.4 mg (*0.15 mg/kg × 62.6 kg*) of dexamethasone IV following the lumbar puncture [6]. Although steroid therapy is known to diminish mitogen responsiveness, no data is available to indicate, a steroid dose needed to shift responsiveness from the “normal” to the “abnormal” range [7].

The recommended trough level of vancomycin in CSF for treating gram-positive shunt infection is between 5–10 mg/L at the 48^th^ hour [8]. However, Reiter et al. showed that the CSF vancomycin level after IV administration of the drug was inadequate for eradication of the organisms in premature infants [9]. Similarly, in our adolescent patient, the vancomycin trough level remained below the target level after IV administration, prompting the IVT administration.

Adult case reports describing intraventricular vancomycin suggest benefit in patients with BA refractory to standard management or with rupture into the CSF space [10]. Advantages include bypassing the blood brain barrier and achieving higher therapeutic concentrations intrathecally.

In pediatric patients, intraventricular antibiotic administration is mostly to target gram-negative meningitis [11]. Whereas existing evidence in the pediatric age group demonstrates intraventricular vancomycin used principally for ventriculoperitoneal shunt infections, all of which were in neonates or infants. Further indications include meningitis and ventriculitis [3].

Therapeutic regimens are highly heterogeneous, with an approximate range of 2–21 days, and empiric doses of 2–20 mg daily are most commonly used [3]. Previous studies suggest that the initial dosing of intraventricular vancomycin for pediatric patients should be individualized and can be adjusted based on the ventricular size and volume [12, 13]. However, there is a lack of sufficient information regarding the pharmacokinetics in the literature for pediatric patients.

The only identified contraindication is hypersensitivity. Adverse effects have been reported only twice so far and include a throbbing headache and elevated leukocyte count in CSF [14].

## Conclusion

To the best of our knowledge, this is the first instance of intraventricular vancomycin used for ventriculitis associated with multiple BAs vs. IVROBA in the pediatric age group. Although our report suggests a potential benefit of intraventricular vancomycin in pediatric BA, its possible role in decreasing mortality and morbidity warrants further investigation and validation on a larger cohort, and research focusing on standardizing dosage regimens.

## References

[1] Mameli C, Genoni T, Madia C, Doneda C, Penagini F, Zuccotti G (2019). Brain abscess in pediatric age: a review. Childs Nerv Syst.

[2] Omar AT, Khu KJO (2020). Successful management of intraventricular rupture of pyogenic brain abscess (IVROBA): Systematic review and illustrative case. J Clin Neurosci.

[3] Liu SP, Xiao J, Liu YL (2022). Systematic review of efficacy, safety and pharmacokinetics of intravenous and intraventricular vancomycin for central nervous system infections. Front Pharmacol.

[4] Petti CA, Simmon KE, Bender J (2008). Culture-Negative intracerebral abscesses in children and adolescents from Streptococcus anginosus group infection: a case series. Clin Infect Dis.

[5] Watson M (2012). Streptococcal Intermedius Brain Abscesses In An Adolescent Male With Histoplasma Infection. Pediatric Oncall.

[6] Bodilsen J, D’Alessandris QG, Humphreys H (2024). European society of Clinical Microbiology and Infectious Diseases guidelines on diagnosis and treatment of brain abscess in children and adults. Clin Microbiol Infect.

[7] Bonilla FA (2008). Interpretation of lymphocyte proliferation tests. Ann Allergy Asthma Immunol.

[8] Nagl M, Neher C, Hager J, Pfausler B, Schmutzhard E, Allerberger F (1999). Bactericidal activity of vancomycin in cerebrospinal fluid. Antimicrob Agents Chemother.

[9] Reiter PD, Doron MW (1996). Vancomycin cerebrospinal fluid concentrations after intravenous administration in premature infants. J Perinatol.

[10] Doan N, Nguyen H, Luyuan L, Shabani S, Gelsomino M, Johnson V (2018). Good Outcomes with the Intraventricular Vancomycin Therapy in a Patient with Ruptured Brain Abscesses. Asian J Neurosurg.

[11] Deniz M, Tapısız A, Börcek A, Tezer H (2021). Intraventricular treatment of paediatric meningitis due to extensively drug-resistant Gram-negative bacteria: two case reports and review of the literature. J Chemother.

[12] Parasuraman JM, Albur M, Fellows G, Heep A (2018). Monitoring intraventricular vancomycin for ventriculostomy access device infection in preterm infants. Childs Nerv Syst.

[13] Bafeltowska JJ, Buszman E, Mandat KM, Hawranek JK (2004). Therapeutic vancomycin monitoring in children with hydrocephalus during treatment of shunt infections. Surg Neurol.

[14] Ng K, Mabasa VH, Chow I, Ensom MH (2014). Systematic review of efficacy, pharmacokinetics, and administration of intraventricular vancomycin in adults. Neurocrit Care.

